# Feasibility of a social network-based physical activity intervention targeting vocational students

**DOI:** 10.1093/eurpub/ckac131.315

**Published:** 2022-10-25

**Authors:** L Guenther, S Schleberger, CR Pischke

**Affiliations:** Heinrich-Heine-University Duesseldorf, Institute of Medical Sociology, Düsseldorf, Germany

## Abstract

**Background:**

Globally, four of five adolescents do not meet the recommendations for physical activity (PA). This is a public health concern because moving large segments of young adults from inactivity to activity, is essential to reach the global target of a 15% relative reduction of inactivity by 2030 worldwide. This study aimed to pilot a social network-based PA intervention in a sample of young adults enrolled at vocational schools.

**Methods:**

Fourteen students from one vocational school located in Duesseldorf were encouraged to walk 10.000 steps per day over an intervention period of six weeks. In the WALK2gether intervention, students received general information on PA in a Facebook group and were instructed to use the pedometer app Pacer to monitor their individual steps and compare their daily step count with fellow participants. The framework by Thabane et al. (2010) was was taken as a basis for examining the feasibility of the methods and procedures employed and for estimating the magnitude of potential intervention effects.

**Results:**

The WALK2gether intervention turned out to be minimally resource intensive with, in total, 92 hours spent by the study staff on development and implementation. The recruitment rate was 19.2% and loss-to-follow was 28.6%. Descriptively analysed data revealed no noteworthy changes from baseline to follow-up neither in PA nor in other health outcomes, such as body mass index and quality of life. The target population did not interact in the Facebook group, while a moderate interaction with Pacer was noted.

**Conclusions:**

This pilot study and intervention were only partially feasible. Although the results ought to be interpreted with caution due to the small sample size, our results suggest that the target group would rather benefit from a structured PA regimen with regular check-ins and PA counselling, possibly by vocational school teachers, than the very autonomous approach piloted in this study.

**Key messages:**

• Results of this pilot study can inform the development and implementation of future social media-based PA interventions targeting young adults in vocational schools.

• Optimization of the intervention and studies on a larger scale are necessary.

